# Benign Paroxysmal Positional Vertigo

**DOI:** 10.1155/2011/353865

**Published:** 2011-10-17

**Authors:** Stavros Korres, Linda Luxon, Paolo Vannucchi, Bill Gibson

**Affiliations:** ^1^1st ENT Department, Hippokration Hospital of Athens, 114 Vasilissis Sofias Avenu, National University of Athens, 11527 Athens, Greece; ^2^UCL Ear Institute, London, UK; ^3^Department of Surgical Sciences Oto-Neuro-Ophthalmology, 50121 Florence Service of Audiology, University of Florence, Italy; ^4^Department of Surgery/Otolaryngology, The University of Sydney, Australia

Dizziness and vertigo are among the most frequently encountered symptoms in primary care, with benign paroxysmal positional vertigo (BPPV) being the commonest type of vertigo. Its clinical course may vary considerably from a self-treatable to a persisting and/or recurrent disabling problem, with as yet unidentified prognostic factors. Although it is named as such, there are a considerable number of patients who do not perceive it as a benign disease, but rather as an incapacitating condition that restricts their routine activities and has a significant impact on their quality of life [1, 2]. 

*Current Diagnosis and Management*

Until the theories of canalithiasis and cupulolithiasis were reported, the treatment of BPPV had been based on either the avoidance of the provoking positions or habituation. The assumption that the dislodgment of otoconia toward the semicircular canals or the ampulla is the underlying pathophysiological mechanism has led to the development of canalith repositioning procedures (CRPs) [3–6]. Indeed, the successful results attributed to CRPs seem to have verified the respective theories. The careful observation, through the Frenzel glasses or videonystagmography, of the nystagmus provoked by simple changes in the position of the patient's head can usually provide the ability to localize the dislodged otoconia in the ampulla or the lumen of one or more of the six semicircular canals (SCCs) [7]. In some cases diagnosis can also go as far as detecting, for example, that the dislodged otoconia is located in the posterior arm of the horizontal SCC (canalolithiasis) if the nystagmus is geotropic in side positions, or in the anterior arm of the horizontal SCC, either free floating (canalolithiasis) or attached to the cupula (cupulolithiasis), if the nystagmus is apogeotropic [8–10]. The details in the diagnosis that a specialized observer can reach through a noninvasive and simple-to-perform examination are indeed quite amazing, while the observation of nystagmus during CRPs allows speculations on the movement of debris and the appropriate treatment strategy. Finally, the simultaneous or successive insult of multiple canals might be a complex issue in the diagnosis and treatment of BPPV.

*Challenges in Diagnosis and Treatment*

Due to the existence of several and to a large extent unknown contributing factors, BPPV remains a challenging field that is constantly evolving in terms of pathophysiology, clinical manifestation, recovery, treatment, and recurrence. For example, clinicians sometimes encounter atypical and intractable BPPV patients who show frequent relapses or poor response to physical therapy. Anatomic variations, stenoses in the SCC lumen, or multiple clots of particles in the same SCC which cause unpredictable endolymphatic currents can account for some of the “difficult” cases. Another observation with intriguing underlying pathophysiology is the fact that the treatment of BPPV secondary to head trauma is less effective than that of idiopathic BPPV. Several other causes of intractability in BPPV have been reported including osteoporosis [11], trauma [12], position during bed rest [13], diabetes [14], and Ménière's disease [15]. Furthermore, there seems to exist an interesting but poorly understood relationship between migraine and BPPV [16, 17]. Finally the anterior SCC was considered in the past as being free from dislodged otoconia due to its anatomical position, but recent observations have proved that such a BPPV variation, although rare, is indeed possible [18, 19].

The considerable variation in the vertical and torsional contributions to the nystagmus induced by the Dix Hallpike maneuver, especially when considering the anterior and posterior canals, constitutes another pathophysiologic and diagnostic question which cannot be explained solely by the canalolithiasis and cupulolithiasis theories [20]. The role of the interactions between the semicircular canals and the otolith organs in the clinical signs and symptoms, as well as the recovery from BPPV, is still under investigation [21, 22]. Potential involvement of the vestibular nuclei, ganglia, peripheral nerve fibres, and central nervous system vestibular centres is also being studied [23].

Recent high-resolution magnetic resonance imaging (MRI) seems to be able to identify an obliteration of the membranous labyrinth as filling defects of the inner ear fluid spaces, semicircular canal stenosis, and/or a plug of otoconial debris, and morphological abnormalities in the inner ear of patients with intractable BPPV such as fractures, stenoses, filling defects, and a fold of semicircular canals have been reported [24, 25].

BPPV is a frequent disease which is usually resolved spontaneously or by office-based canalith repositioning maneuvers. In some cases, however, it may also be manifested as a persisting and/or recurrent disabling problem. During the last decade, the treatment of these “difficult”, although rare, cases has rendered BPPV a constantly evolving and intriguing subject of investigation. Partly due to its numerous potential variations and partly due to its yet unknown pathophysiological mechanisms and predisposing and prognostic factors, BPPV remains a challenging and promising investigation field.

*Stavros Korres* *Stavros Korres* *Linda Luxon* *Linda Luxon* *Paolo Vannucchi* *Paolo Vannucchi* *Bill Gibson* *Bill Gibson*
